# Biomass-Derived P/N-Co-Doped Carbon Nanosheets Encapsulate Cu_3_P Nanoparticles as High-Performance Anode Materials for Sodium–Ion Batteries

**DOI:** 10.3389/fchem.2020.00316

**Published:** 2020-05-05

**Authors:** Yanyou Yin, Yu Zhang, Nannan Liu, Bing Sun, Naiqing Zhang

**Affiliations:** ^1^State Key Laboratory of Urban Water Resource and Environment, Harbin Institute of Technology, Harbin, China; ^2^Faculty of Science, Center for Clean Energy Technology, School of Mathematical and Physical Science, University of Technology Sydney, Sydney, NSW, Australia; ^3^Harbin Institute of Technology, Academy of Fundamental and Interdisciplinary Sciences, Harbin, China

**Keywords:** sodium-ion batteries, biomass, Cu_3_P, P/N-co-doped carbon, nanosheets

## Abstract

Biomass-derived approaches have been accepted as a practical way for the design of transitional metal phosphides confined by carbon matrix (TMPs@C) as energy storage materials. Herein, we successfully synthesize P/N-co-doped carbon nanosheets encapsulating Cu_3_P nanoparticles (Cu_3_P@P/N-C) by a feasible aqueous reaction followed by a phosphorization procedure using sodium alginate as the biomass carbon source. Cu-alginate hydrogel balls can be squeezed into two-dimensional (2D) nanosheets through a freeze–drying process. Then, Cu_3_P@P/N-C was obtained after the phosphorization procedure. This rationally designed structure not only improved the kinetics of ion/electron transportation but also buffered the volume expansion of Cu_3_P nanoparticles during the continuous charge and discharge processes. In addition, the 2D P/N co-doped carbon nanosheets can also serve as a conductive matrix, which can enhance the electronic conductivity of the whole electrode as well as provide rapid channels for electron/ion diffusion. Thus, when applied as anode materials for sodium-ion batteries, it exhibited remarkable cycling stability and rate performance. Prominently, Cu_3_P@P/N-C demonstrated an outstanding reversible capacity of 209.3 mAh g^−1^ at 1 A g^−1^ after 1,000 cycles. Besides, it still maintained a superior specific capacity of 118.2 mAh g^−1^ after 2,000 cycles, even at a high current density of 5 A g^−1^.

## Introduction

In recent years, lithium-ion batteries (LIBs) have been widely applied from portable electronic devices to electric vehicles (Zou et al., 2017, 2019; Qiu et al., 2019). However, the shortage of lithium sources limited the further development of LIBs (Wang et al., 2018a; Wu C. et al., 2018). Over the past few years, sodium-ion batteries (SIBs), owing to their low cost of production and earth-abundant sodium sources, show tremendous potential as a promising replacement of LIBs for large-scale energy storage applications (Kundu et al., 2015; Larcher and Tarascon, 2015; Zhang et al., 2016, 2018; Song et al., 2018; Xiao et al., 2018, 2020). However, when compared with LIBs, SIBs are still an immature technology that is confronted with a lot of challenges, such as low specific energy, poor cycleability, and low power density (Wessells et al., 2011; Lotfabad et al., 2014; Li and Zhou, 2018). Anode, as the main component of SIBs, has a great influence on the overall electrochemical performance. In recent years, red phosphorus has been regarded as one of promising SIB anode materials owing to its comparatively low redox potential (~0.4 V vs. Na/Na^+^) and extremely high theoretical specific capacity (2,596 mAh g^−1^) (Kim et al., 2013; Qian et al., 2013; Zhou et al., 2017; Hu et al., 2018; Wu Y. et al., 2018). However, the low electrical conductivity (~10^−14^ S cm^−1^) and the huge volume expansion (~490%) during the continuous Na^+^ insertion/extraction process make red phosphorus suffer from inferior cycling stability and rate performance (Sun et al., 2014; Wang et al., 2018b). Fortunately, forming transition metal phosphides (TMPs) by combining red phosphorus with conductive transition metals has been proven to be an efficient way to enhance the electronic conductivity and reduce the volume change of phosphorus-based anode materials (Fullenwarth et al., 2014; Pramanik et al., 2015; Fan et al., 2016; Wang X. et al., 2017; Zhang et al., 2017; Liu et al., 2018).

Transitional metal phosphides (TMPs, M = Fe, Cu, Co, etc.) have drawn tremendous attention because of their high specific capacity and safe operating potential (Kim et al., 2013; Qian et al., 2013; Sun et al., 2014; Zhou et al., 2017; Hu et al., 2018; Wu Y. et al., 2018). Particularly, copper phosphide-based anode materials for SIBs have a low reduction potential (0.015–0.4 V *vs*. Na^+^/Na) and comparatively high specific capacity (Fan et al., 2016; Kong et al., 2018). However, similar to other conversion-type SIB anode materials, the volume expansion during the charge/discharge process has not been entirely overcome, and its diffusion kinetics is comparatively weak (Ge et al., 2017; Miao et al., 2017; Wang J. et al., 2017).

Fortunately, several effective strategies revealed promising potential ability in boosting the sodium storage performance of TMP anode materials. For example, constructing nanostructured materials, such as nanospheres and nanoparticles, can not only improve the reaction kinetics by shortening the diffusion distance of Na ions within the solid state but also relieve the mechanical strain generated by the large volume change during the conversion reaction (Xu et al., 2012; Ma et al., 2018). In addition, combining TMPs with conductive carbon matrices can also buffer the huge volume expansion and enhance the electronic conductivity of the electrode, thus resulting in better sodium storage performance (Qian et al., 2014; Li et al., 2017; Zhang et al., 2019). For instance, Cu_3_P/reduced graphene oxide nanocomposite synthesized by Tong et al. showed superior cycling stability and good rate capability (Liu et al., 2016). The carbon-confined Cu_3_P nanoparticles prepared by Zhou et al. exhibited superior cycling stability with a high capacity of 159 mAh g^−1^ at 1.0 A g^−1^ over 100 cycles (Kong et al., 2018). Although much progress has been achieved by reducing the particle size of Cu_3_P as well as introducing conductive carbon, it still remains a major challenge to develop a scalable and inexpensive method for practical application.

Biomass-derived carbon, profiting from its economy, environmental benignity, and sustainability, has attracted increasing attention (Moreno et al., 2014; Wu et al., 2017). Among them, sodium alginate has high availability and biodegradability, is non-toxic, and of low price (Comaposada et al., 2015). In particular, sodium alginate can cross-link with di- or trivalent ions (Marcos et al., 2016), thus making it a proper carbon source to synthesize TMP anode materials combined with conductive carbon matrices.

Herein, we present the preparation of Cu_3_P nanoparticles encapsulated in P/N-co-doped carbon nanosheets (Cu_3_P@P/N-C) through a feasible aqueous reaction followed by a phosphorization procedure. Cu_3_P nanoparticles are well-dispersed and encapsulated in two-dimensional (2D) carbon nanosheets, which can not only buffer the large volume expansion but also prevent the agglomeration of Cu_3_P nanoparticles, thereby maintaining the integrity of the whole electrode. Furthermore, the 2D carbon nanosheet structure can shorten the Na^+^ diffusion path, provide more active sites of Na^+^, as well as enhance the electronic conductivity of the entire electrode. Benefiting from these advantages mentioned above, Cu_3_P@P/N-C exhibited a long cycle life and outstanding rate performance when applied as anode for SIBs. Cu_3_P@P/N-C anode materials demonstrated a long cycle life (209.3 mAh g^−1^ at 1 A g^−1^ after 1,000 cycles) and excellent rate performance (118.2 mAh g^−1^ even at a high current density of 5 A g^−1^ after 2,000 cycles).

## Experimental Section

### Materials

All materials in the experiment were used without further purification. Cu(NO_3_)_2_·3H_2_O (ACS, 98.0–102.0%) was purchased from Aladdin. Sodium alginate (AR) was purchased from Aladdin. Red phosphorus (AR) was purchased from Kermel. Argon gases were supplied in cylinders by Qinghuaqiti with 99.999% purity.

### Preparation of Cu-Alginate Gel

Sodium alginate (1.0 g) was dispersed in 50 ml distilled water to form an aqueous solution. Cu(NO_3_)_2_·3H_2_O (2.0 g) was also dispersed in 50 ml distilled water to form an aqueous solution. Then, the sodium alginate aqueous solution was dropped slowly by a disposable plastic dropper into Cu(NO_3_)_2_·3H_2_O aqueous solution to form Cu-alginate hydrogel under magnetic stirring at room temperature. The obtained hydrogel was separated from the solution after 6 h and washed with deionized water several times. The as-prepared Cu-alginate hydrogel was frozen by liquid nitrogen and then dried through freeze–drying for 24 h to obtain Cu-alginate aerogel.

### Preparation of Cu_3_P@P/N-C

In a typical synthesis of Cu_3_P@P/N-C, Cu-aerogel was kept at 300°C for 1 h at a heating rate of 2°C min^−1^ under air atmosphere. After cooling to room temperature, the CuO@C nanosheets aerogel was collected (Figure S1). One hundred milligrams of the obtained CuO@C nanosheet aerogel and 200 mg red phosphorus were put into two separate ceramic boats in a tube furnace and then were heated to 800°C for 2 h under Ar atmosphere with a heating rate of 5°C min^−1^ to produce Cu_3_P@P/N-C. After cooling to room temperature, the sample was washed with deionized water three times by centrifugation and dried at 60°C in vacuum oven for 12 h.

### Morphology and Structural Characterization

The morphology of the obtained samples was characterized by a field-emission scanning electron microscope (Hitachi Limited SU-8010) and transmission electron microscopy (JEOL-2100FS). The X-ray diffraction (XRD) pattern was determined by PANalytical X'Pert PRO (PANalytical X'Pert PRO, monochromated Cu Kα radiation 40 mA, 40 kV) to characterize the crystal structure. X-ray photoelectron spectroscopy (XPS) was performed with a Thermo Fisher Scientific K-Alpha (Fisher Scientific Ltd., Nepean, ON).

### Electrochemical Measurements

The anode slurry was prepared by mixing 70 wt% active materials, 20 wt% Super-P, and 10 wt% polyvinylidene fluoride (PVDF) by a high-speed electric agitator for 12 h. The slurry was pressed onto a cleaned copper foil by a doctor-balding method and dried in a vacuum oven at 80°C for 12 h. The performance of the SIBs was tested using standard 2032-type coin cells in an argon-filled glove box. The separator was glass fiber (GF/D) from Whatman, and sodium foils were used as the counter and reference electrodes. The electrolyte was 1.0 M NaClO_4_ in diethylene glycol dimethyl ether (Diglyme). The active material loading of the electrode was 0.5–0.8 mg cm^−2^. The cells were galvanostatically charged and discharged over a cutoff voltage window of 0.01–3.00 V at room temperature on a battery test system (Shenzhen Neware Electronic Co., China). Cyclic voltammetry behavior was studied by the CHI 650d electrochemical workstation at a scan rate of 0.1 mV s^−1^.

## Results and Discussion

**Scheme 1:**
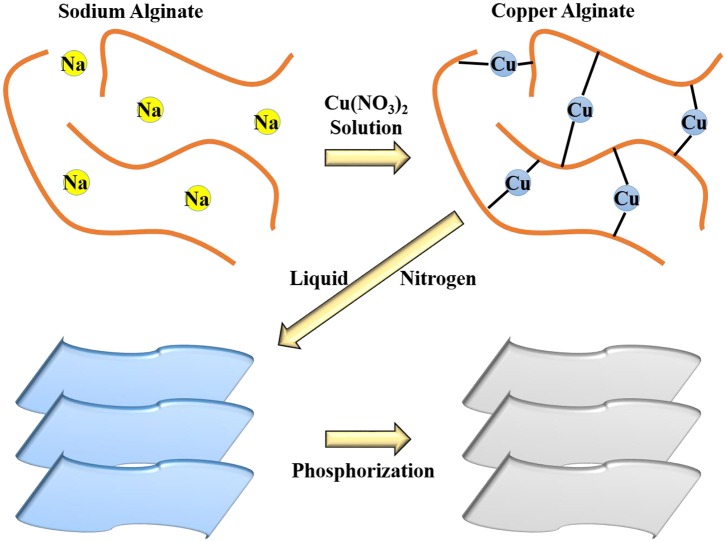
Schematic illustration of the synthesis process of Cu_3_P@P/N-C.

The carbon source we chose is sodium alginate. Sodium alginate is a natural polysaccharide extracted from brown seaweeds and some kinds of bacteria, which consists of a linear copolymer of (1–4)-linked β-d-mannuronic acid (M) and α-l-guluronic acid (G) in alternating blocks. Sodium alginate has high availability and biodegradability and low price. Its aqueous solution has a high viscosity and is non-toxic, which makes it widely useful as food thickeners, stabilizers, emulsifiers, etc. (Comaposada et al., 2015; Zou et al., 2018). In particular, sodium alginate can cross-link with di- or trivalent ions to form the uniform, transparent, water-insoluble, and thermo-irreversible gels at room temperature (Marcos et al., 2016). Scheme 1 exhibits the typical synthesis of Cu_3_P@P/N-C. Sodium alginate aqueous solution was added dropwise into Cu(NO_3_)_2_·3H_2_O aqueous solution to form Cu-alginate hydrogel balls. During the freezing process by liquid nitrogen, subsequently, the growth of the ice crystals squeezed the Cu-alginate macromolecules into 2D nanosheets. Then, the sample was dried by freeze drying. In the end, after the phosphorization procedure, Cu_3_P@P/N-C was obtained.

**Figure 1:**
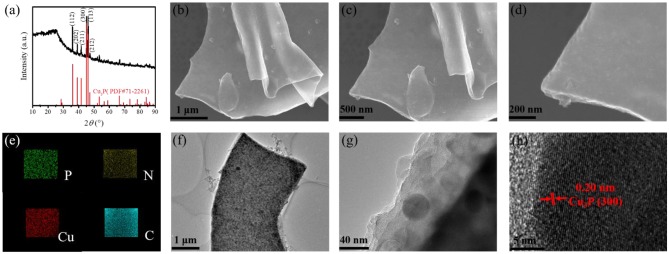
**(a)** X-ray diffraction (XRD) pattern of Cu_3_P@P/N-C. **(b–d)** SEM images, **(e)** energy-dispersive X-ray (EDX) mappings, **(f,g)** transmission electron microscopy (TEM) images, and **(h)** high-resolution TEM (HRTEM) image of Cu_3_P@P/N-C.

As shown in Figure 1a, the crystal structure and composition of the as-prepared samples were first tested by XRD measurements. There are several diffraction peaks in the XRD pattern of Cu_3_P@P/N-C at 36.0, 39.1, 41.6, 45.1, 46.1, and 47.3°. These peaks can be indexed to the (112), (202), (211), (300), (113), and (212) lattice planes of Cu_3_P crystalline (PDF#71-2261), matching well with a formerly reported study (Wang R. et al., 2018). In addition, there is a broad peak at around 24.7°, corresponding to the (002) plane of amorphous carbon. There are no other crystalline phases observed, which suggests that the as-prepared Cu_3_P@P/N-C has high purity.

The morphologies of the Cu-alginate aerogel and Cu_3_P@P/N-C were investigated by scanning electron microscopy (SEM). Figure S2 exhibits the typical 2D nanosheet morphology of Cu-alginate aerogel. And as shown in Figures 1b–d, the 2D nanosheet morphology remained well after the phosphorization procedure. Figure 1e displays the typical energy-dispersive X-ray (EDX) mappings of Cu_3_P@P/N-C, where P, N, Cu, and C elements were observed, suggesting the homogeneous distribution of these several elements. The distribution of P element was uniform, indicating that P not only came from Cu_3_P but also doped in the carbon nanosheets. The typical 2D nanosheet morphology was also confirmed by the transmission electron microscopy (TEM) image of Cu_3_P@P/N-C (Figures 1f, g). The Cu_3_P nanoparticles with sizes of around 30 nm distributed uniformly as well as encapsulated in P/N-co-doped carbon nanosheets, which can not only provide rapid diffusion channels for the electron/ion but also prevent Cu_3_P nanoparticles from agglomeration. Figure 1h shows the high-resolution transmission electron microscopy (HRTEM) image in which the lattice fringes can be observed distinctly. The distance of these lattice fringes is 0.20 nm, matching well with the Cu_3_P crystalline (300) lattice plane (Liu et al., 2016), verifying the existence of the Cu_3_P nanoparticles.

**Figure 2:**
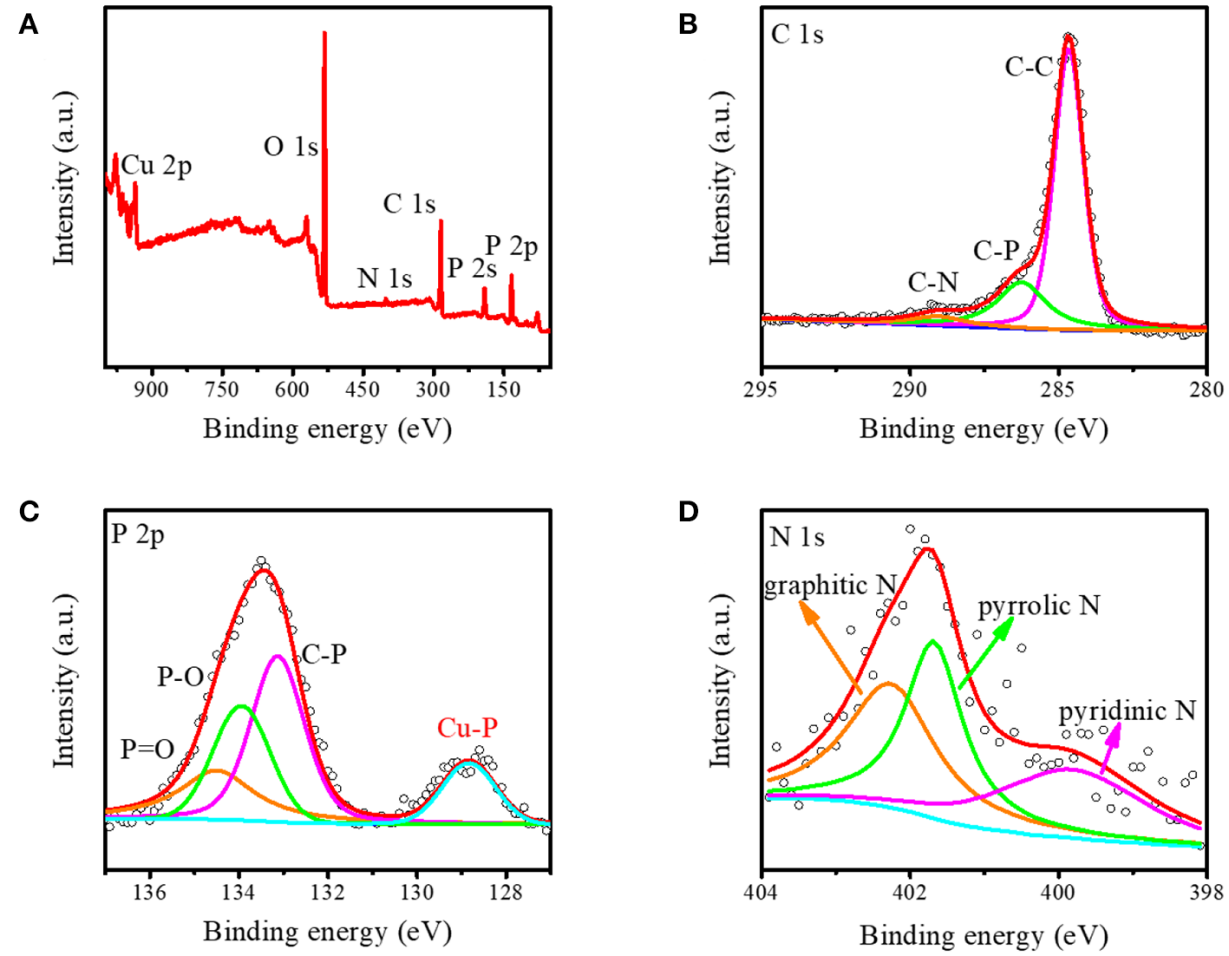
**(A)** The integrated X-ray photoelectron spectroscopy (XPS) spectrum and the corresponding XPS spectrum of **(B)** carbon, **(C)** phosphorus, and **(D)** nitrogen for Cu_3_P@P/N-C.

Furthermore, the X-ray photoelectron energy spectra (XPS) measurement was applied to study the detailed chemical states of Cu_3_P@P/N-C. As shown in Figure 2A, the signals of the C, N, O, P, and Cu elements were observed obviously without other impurities. The element O may come from the absorbed O species in the air. The N element came from the nitrate radical of Cu(NO_3_)_2_·3H_2_O, which was not completely removed during the washing process. The C 1s spectrum in Figure 2B could be attributed to three peaks at 284.6, 286.3, and 289.1 eV. The major peak at approximately 284.6 eV was attributed to graphitic carbon. The other two peaks at 286.3 and 289.1 eV were fitted by carbon bonding with phosphorus and nitrogen, respectively, which can manifest the co-doping of both P and N atoms into the carbon nanosheets. The P2p spectrum in Figure 2C indicates the P chemical states in Cu_3_P@P/N-C. The P2p spectrum could be fitted into four peaks at 129.9, 134.2, 135.1, and 135.6 eV. The peak at 129.9 eV was attributed to the P in Cu_3_P. The two peaks at 135.1 and 135.6 eV were ascribed, respectively, to P–O and P=O bonds. And the peak at 134.2 eV could be ascribed to the C–P bond, corresponding to the C 1s bonding peak at 286.3 eV. The N 1s spectrum (Figure 2D) can be fitted into three component peaks at 399.4, 401.8, and 402.3 eV, which can be assigned to pyridinic nitrogen, pyrrolic nitrogen, and graphitic nitrogen, respectively. This could further confirm that both P and N are doped into the as-prepared carbon nanosheets.

**Figure 3:**
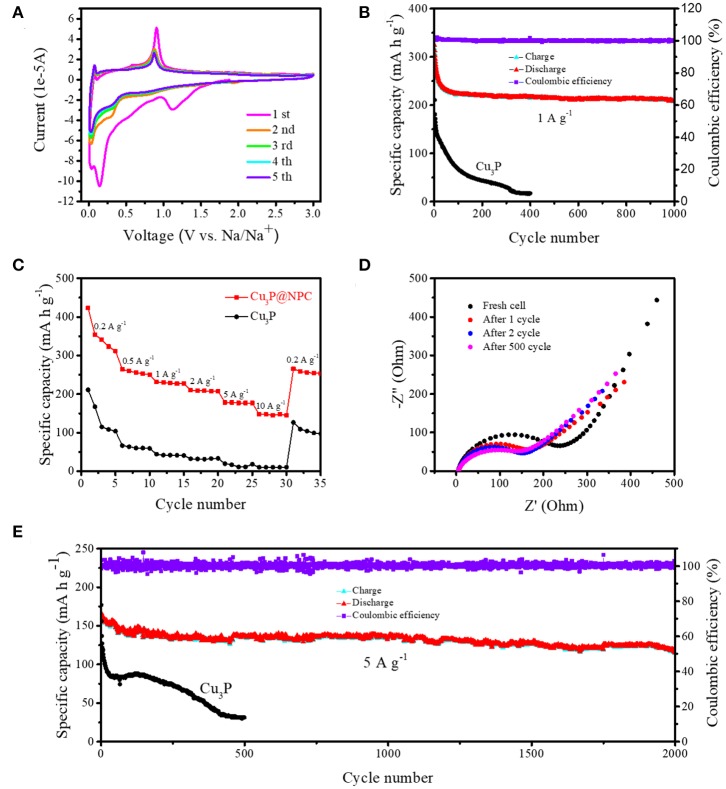
**(A)** Cyclic voltammetry (CV) curves of Cu_3_P@P/N-C at a scan rate of 0.1 mV s^−1^ in the initial five cycles. **(B)** Cycling performance at a current density of 1 A g^−1^ and **(C)** rate performance of Cu_3_P@P/N-C and pure Cu_3_P. **(D)** Electrochemical impedance spectroscopy (EIS) curves of Cu_3_P@P/N-C at different cycles during the first 500 cycles. **(E)** Cycling performance at a current density of 5 A g^−1^ of Cu_3_P@P/N-C and pure Cu_3_P.

The electrochemical performance of Cu_3_P@P/N-C was measured in detail as anode materials for SIBs. First, its electrochemical reaction was studied by cyclic voltammetry (CV) measurements from 0.01 to 3 V at a scan rate of 0.1 mV s^−1^ (Figure 3A). In the first cathodic process of the CV curves of Cu_3_P@P/N-C, there was a reduction peak at around 1.1 V, which can be associated with side reactions [the irreversible decomposition of the electrolyte and the formation of the solid electrolyte interface (SEI) layer on the electrode surface] (Zhu et al., 2019). Then, a strong reduction peak appeared between 0.07 and 0.4 V, which could be ascribed to the reaction of Cu_3_P with sodium. In the anodic scan process, the reverse of this reaction formed a distinct oxidation peak at around 0.9 V, as the following equation with the release of sodium ions shows:

(1) $$\begin{array}{r} \text{N}{\text{a}}_{3}P\;+3Cu\to Cu_{3}P\;+\;3Na^{+} \end{array}$$

The CV profiles from the second to the fifth cycles matched well, indicating the highly reversible and stable cycling performance of Cu_3_P@P/N-C as SIB anode materials.

Figure 3B shows the galvanostatic cycling performance of the Cu_3_P@P/N-C electrode under 1.0 A g^−1^ (0.01 and 3.0 V vs. Na^+^/Na). Pure Cu_3_P was also synthesized by the phosphorization of commercial Cu powder using red phosphorus as a P source for comparison (as shown in Figures S3–S5). Compared with pure Cu_3_P, the Cu_3_P@P/N-C electrode exhibited better electrochemical performance, maintaining an excellent reversible capacity of 209.3 mAh g^−1^ at 1.0 A g^−1^ after 1,000 cycles. The charge–discharge profiles of the initial three cycles of Cu_3_P@P/N-C are shown in Figure S6. The initial discharge and charge capacities were 1,029.6 and 562.3 mAh g^−1^; thus, the initial coulombic efficiency was 54.6%. This capacity loss was mainly caused by the irreversible formation of the SEI layer. Additionally, to estimate the sodium storage performance of the Cu_3_P@P/N-C electrode at a high rate, a high current density at 5.0 A g^−1^ was chosen. An outstanding cycling performance of 118.2 mAh g^−1^ is maintained (Figure 3E), even after 2,000 cycles. As shown in Table S1, Cu_3_P@P/N-C exhibited superior cycling performance and excellent rate performance. In sharp contrast to Cu_3_P@P/N-C, pure Cu_3_P had rapid capacity loss, resulting in only 44.0 mAh g^−1^ at 1.0 A g^−1^ after 200 cycles and 30.6 mAh g^−1^ at 5.0 A g^−1^ after 500 cycles. Thereby, the superior cycling performance of Cu_3_P@P/N-C reflects their structural stability as anode materials for SIBs. The SEM images and the EDX mappings of the selected area of Cu_3_P@P/N-C after 500 cycles are exhibited in Figures S7, S8. The typical nanosheet morphology of Cu_3_P@P/N-C can still be observed obviously, indicating its superior stability.

Electrochemical impedance spectroscopy (EIS) was investigated to further understand the cycling performance of Cu_3_P@P/N-C. As shown in Figure 3D, the semicircle in the high-frequency region corresponded to the charge transfer resistance of the electrode. The sloped line in the low-frequency region is attributed to the Warburg impendence. After the first cycle, the semicircle became much smaller, which could be explained by the formation of the SEI layer. The semicircles overlaid well after the second cycle, indicating the excellent cycling stability of Cu_3_P@P/N-C.

Figure 3C shows the rate performance of the Cu_3_P@P/N-C and pure Cu_3_P electrodes. The current densities were selected from 0.2 to 10 A g^−1^. The reversible specific capacities acquired were of 311.8, 250.3, 226.8, 207.2, 177.1, and 144.8 mAh g^−1^ at the current densities of 0.2, 0.5, 1, 2, 5, and 10 A g^−1^, respectively. When the current density was set back to 0.2 A g^−1^, the reversible specific capacity remained at 265.2 mAh g^−1^, suggesting the outstanding rate performance. On the contrary, pure Cu_3_P showed reversible specific capacities of only 104.0, 59.1, 41.0, 33.4, 18.1, and 10.2 mAh g^−1^ at the current densities of 0.2, 0.5, 1, 2, 5, and 10 A g^−1^, respectively, which were far inferior to those of Cu_3_P@P/N-C. The difference between these two materials could be ascribed to the introduction of P/N-co-doped carbon nanosheets, which could enhance the electron transfer during the discharge/charge process.

**Figure 4:**
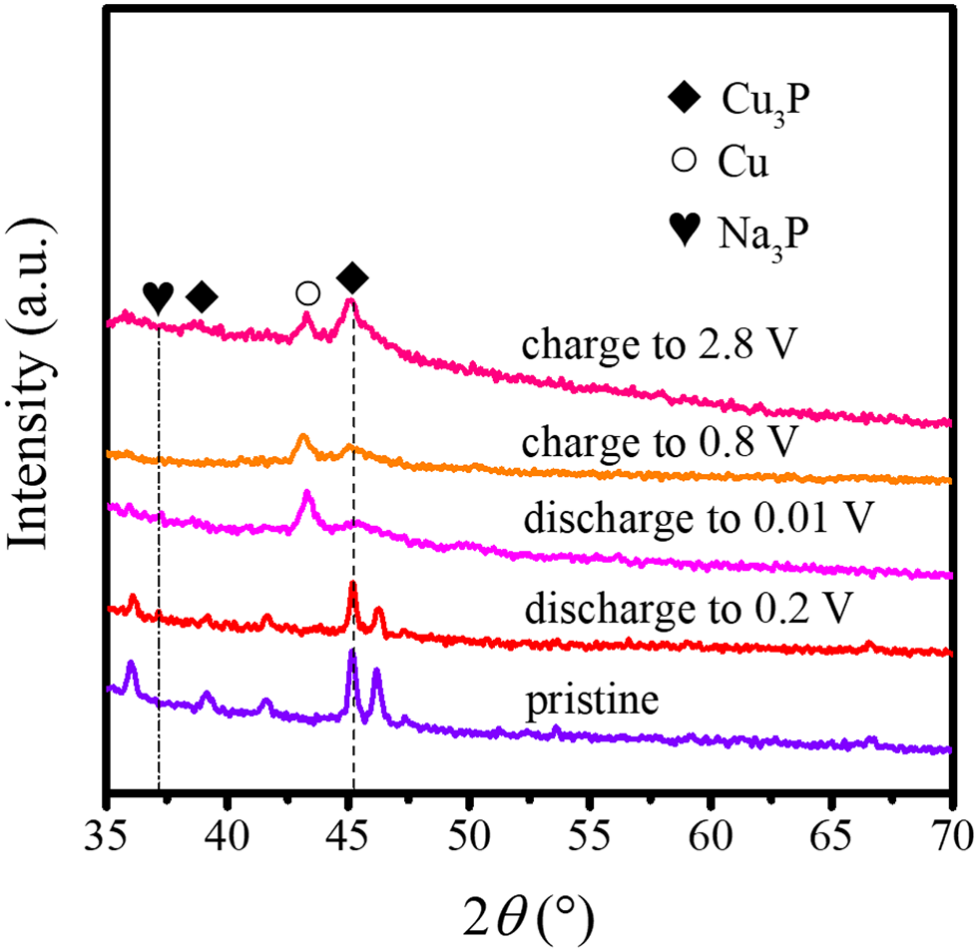
*Ex situ* XRD patterns of Cu_3_P@P/N-C at different states during charge and discharge processes.

To further understand the electrochemical reaction process of the Cu_3_P@P/N-C electrode, we disassembled the cells for the *ex situ* XRD measurement at different states during the charge and discharge processes. As shown in Figure 4, the pristine Cu_3_P@P/N-C electrode only exhibited diffraction peaks of Cu_3_P. During the discharge process, the primary diffraction peak of Cu_3_P at around 45.1° corresponding to the (300) plane gradually receded and almost disappeared when completely discharged to 0.01 V. At the same time, a peak at about 43.5° came out, originating from the generation of Cu. In addition, a new peak came out at about 37° corresponding to the (103) plane of Na_3_P. During the charging process, the above composition change reversed. Such observations can identify the reversible sodiation/desodiation process of Cu_3_P conducting by conversion mechanism as in the following equation (Fan et al., 2016):

(2) $$\begin{array}{r} \text{C}{\text{u}}_{3}P\;+\;3Na↔Na_{3}P\;+\;3Cu \end{array}$$

This conversion mechanism was consistent with the CV measurements.

## Conclusion

In summary, through a feasible aqueous reaction at room temperature followed by a phosphorization procedure, we have successfully fabricated biomass-derived P/N-co-doped carbon nanosheets encapsulating Cu_3_P nanoparticles as high-performance anode materials for sodium-ion batteries. Given the help of the 2D P/N-co-doped carbon nanosheets, the Cu_3_P nanoparticles could be well-encapsulated, thus being prevented from agglomeration. When applied as anode materials, superior cycling stability and excellent rate performance were exhibited. Prominently, Cu_3_P@P/N-C exhibited an outstanding reversible capacity of 209.3 mAh g^−1^ at 1 A g^−1^ after 1,000 cycles. In particular, an excellent specific capacity of 118.2 mAh g^−1^ could be maintained after 2,000 cycles, even at an ultrahigh current density of 5 A g^−1^. This remarkable electrochemical performance is mainly attributed to the rational design of the 2D P/N-co-doped carbon nanosheet structure, which could buffer the volume change of Cu_3_P nanoparticles as well as improve the electron/ion transport kinetics during the Na^+^ insertion/extraction process. Additionally, the 2D P/N-co-doped carbon nanosheets also could serve as a conductive matrix, which could enhance the electronic conductivity of the electrode. This feasible and facile preparation approach could be further developed to the synthesis of a variety of 2D P/N-co-doped carbon nanosheets encapsulating TMP electrodes for energy storage devices.

## Data Availability Statement

All datasets generated for this study are included in the article/Supplementary Material.

## Author Contributions

YY and NZ contributed to the conception and design of the study. YY organized the database. YY, YZ, and NL performed the statistical analysis. YY wrote the manuscript. BS and NZ helped perform the analysis with constructive discussions. All authors contributed to manuscript revision, read, and approved the submitted version.

## Conflict of Interest

The authors declare that the research was conducted in the absence of any commercial or financial relationships that could be construed as a potential conflict of interest.
