# Supplementary material for: Biomass-Derived P/N-Co-Doped Carbon Nanosheets Encapsulate Cu3P Nanoparticles as High-Performance Anode Materials for Sodium–Ion Batteries
Source: Front Chem. 2020 May 5;8:316. doi: 10.3389/fchem.2020.00316 (PMC7216970; doi:10.3389/fchem.2020.00316)
Supplement: Supplementary file 1 [file Data_Sheet_1.docx]

Supplementary Material

**Biomass derived P/N-codoped carbon nanosheets encapsulate Cu_3_P nanoparticles as high-performance anode materials for sodium-ion batteries**

**Yanyou Yin,^1^ Yu Zhang,^1^Nannan Liu****,^1^ Bing Sun,^2*^ Naiqing Zhang,^1,3*^**

1 State Key Laboratory of Urban Water Resource and Environment, Harbin Institute of Technology, Harbin 150001 (China)

2 Center for Clean Energy Technology, School of Mathematical and Physical Science, Faculty of Science, University of Technology Sydney, Sydney, NSW 2007, Australia

3 Academy of Fundamental and Interdisciplinary Sciences, Harbin Institute of Technology, Harbin 150001 (China)

*** Correspondence:**

Naiqing Zhang [znqmww@163.com](mailto:znqmww@163.com)
Bing Sun bing.sun@uts.edu.au

**SUPPORTING INFORMATION CONTENT**

**Figure S1**. XRD patterns of CuO@C.

**Figure S2**. SEM images of Cu-alginate aerogel.

**Figure S3**. XRD patterns of pure Cu_3_P.

**Figure S4**. SEM images of pure Cu_3_P.

**Figure S5**. EDX mappings of selected area of pure Cu_3_P.

**Figure S6**. Charge-discharge profiles of the initial three cycles of Cu_3_P@P/N-C.

**Figure S7**. SEM images of Cu_3_P@P/N-C after 500 cycles.

**Figure S8**. EDX mappings of selected area of Cu_3_P@P/N-C after 500 cycles.

**Table 1**. A brief summary of the typical TMPs based anode materials for SIBs.


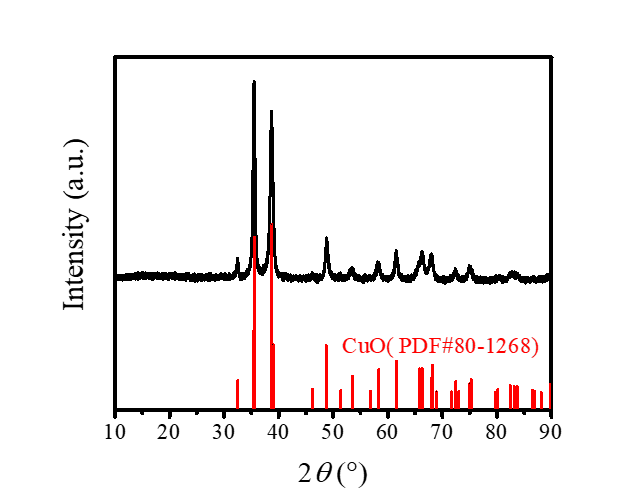


**Figure S1**. XRD pattern of CuO@C.


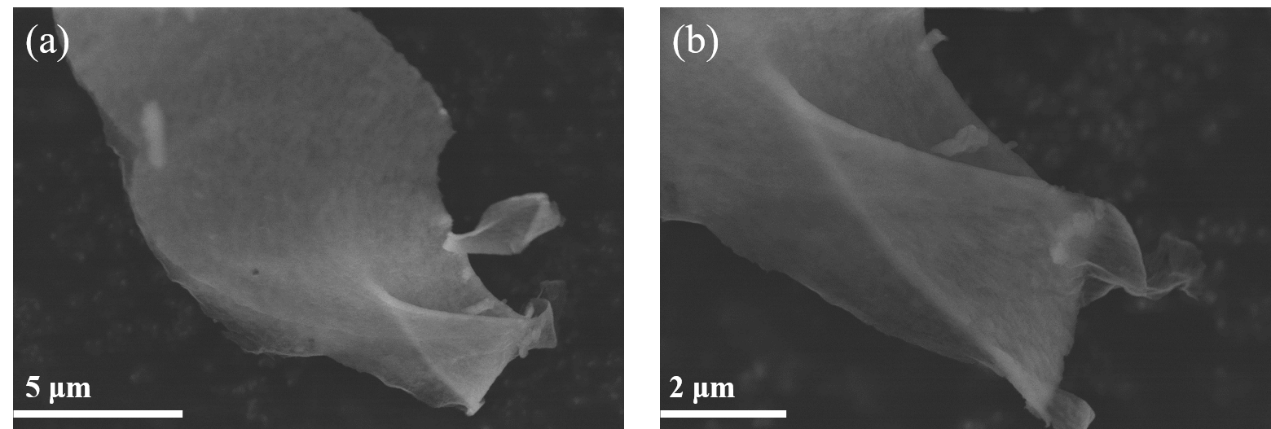


**Figure S2**. SEM images of Cu-alginate aerogel.


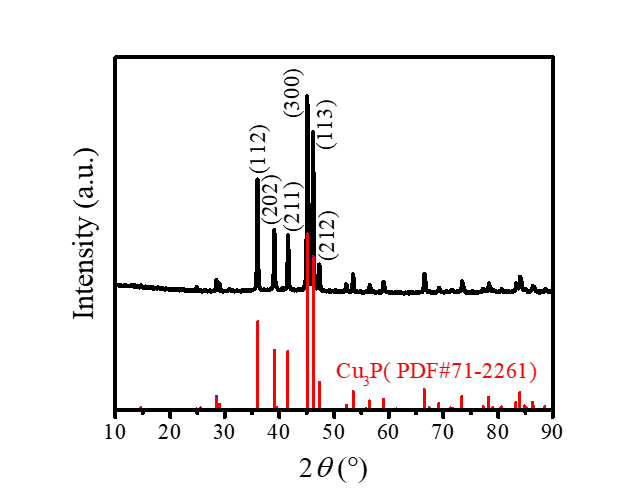


**Figure S3**. XRD patterns of pure Cu_3_P.


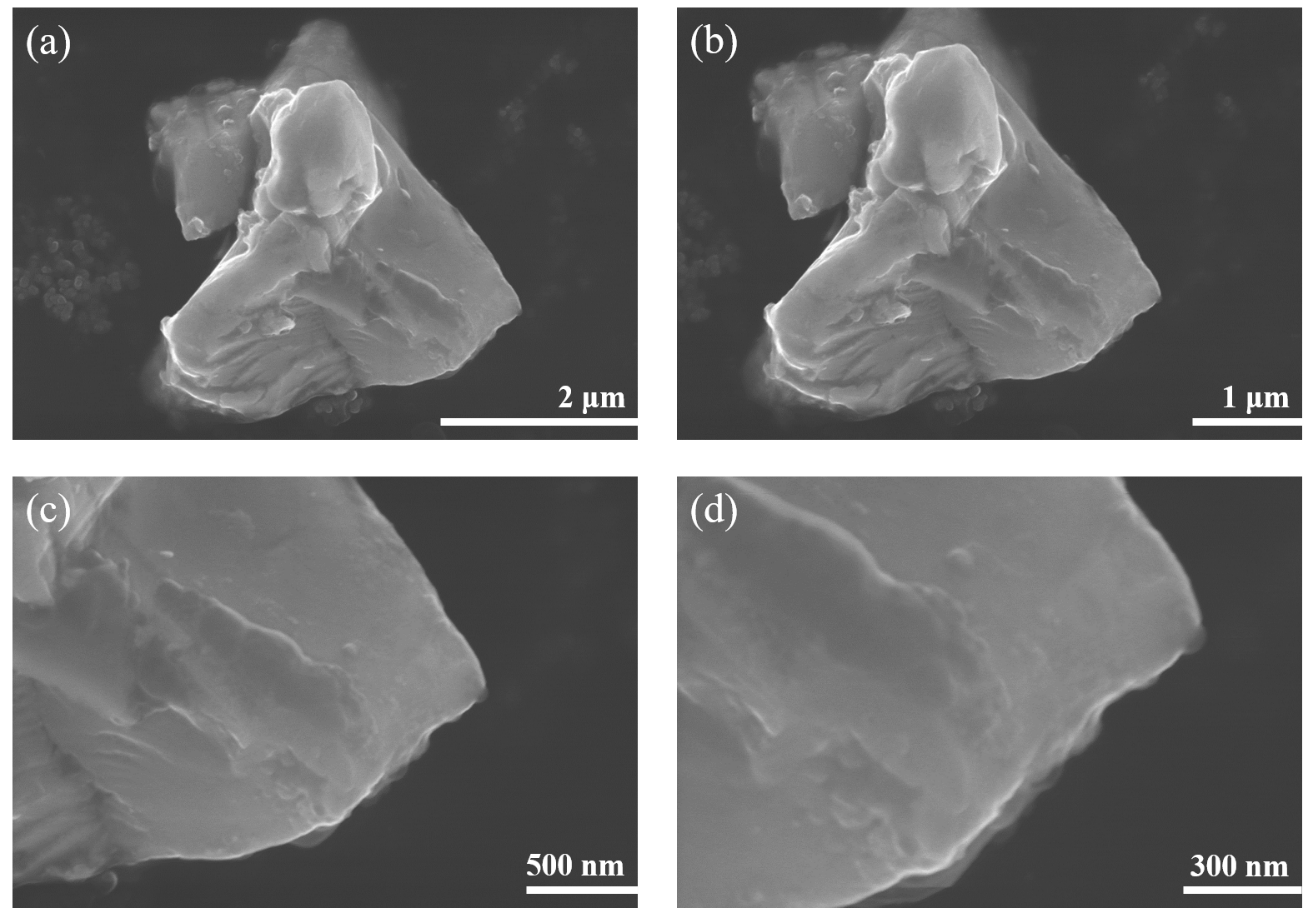


**Figure S4**. SEM images of pure Cu_3_P.


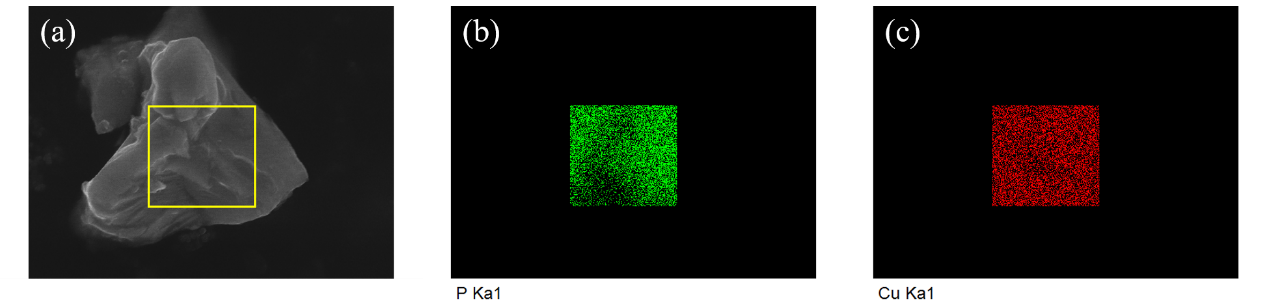


**Figure S5**. EDX mappings of selected area of pure Cu_3_P.


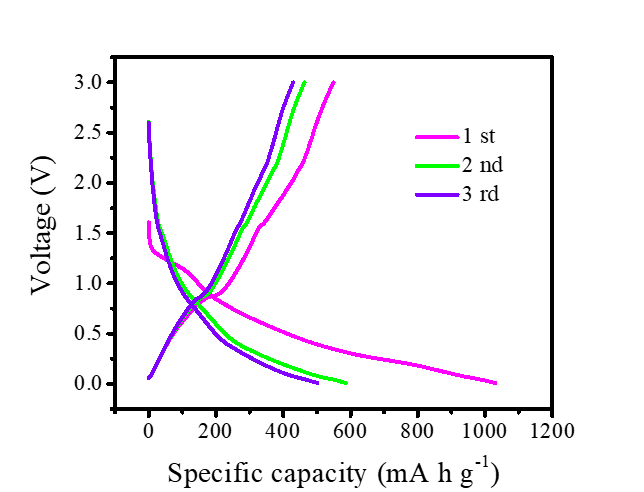


**Figure S6**. Charge-discharge profiles of the initial three cycles of Cu_3_P@P/N-C.


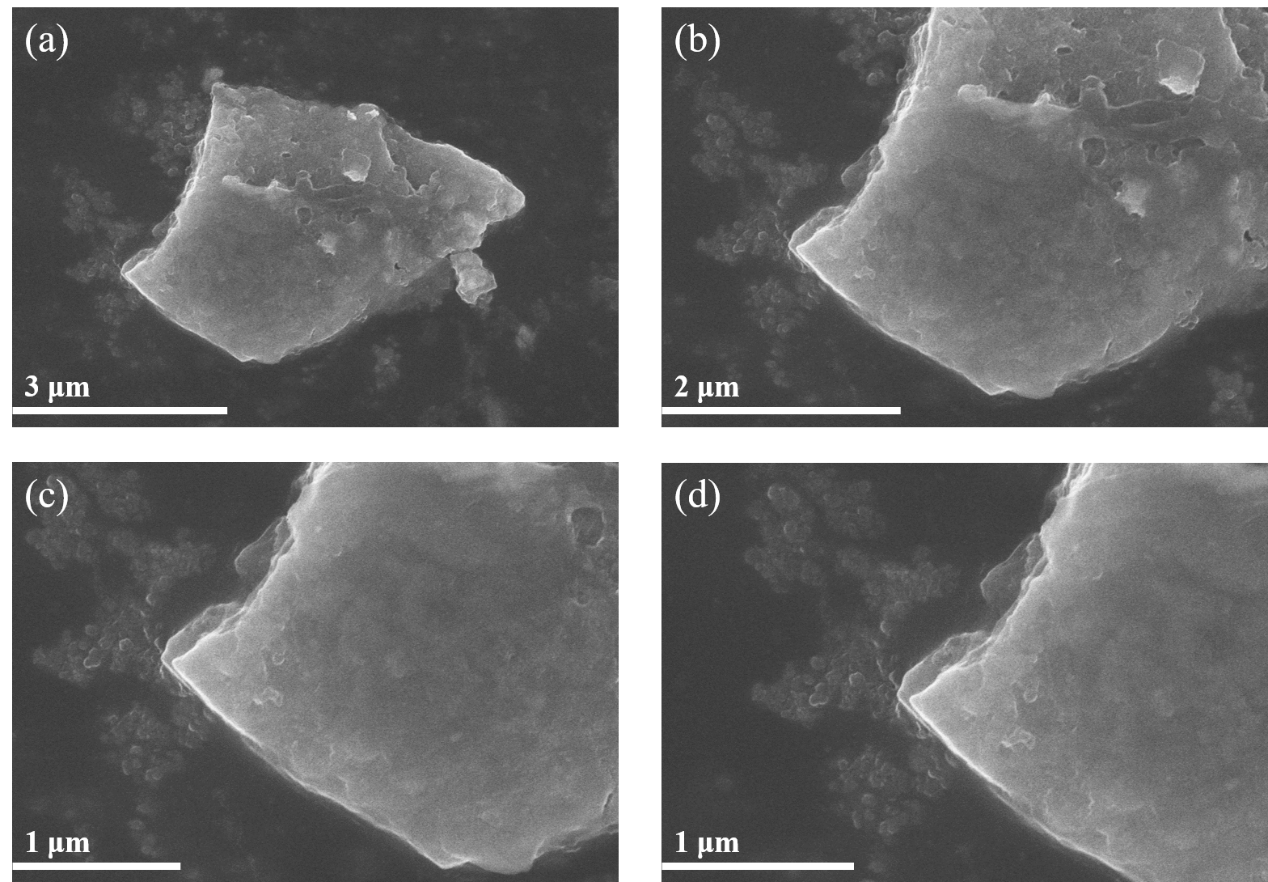


**Figure S7**. SEM images of Cu_3_P@P/N-C after 500 cycles.


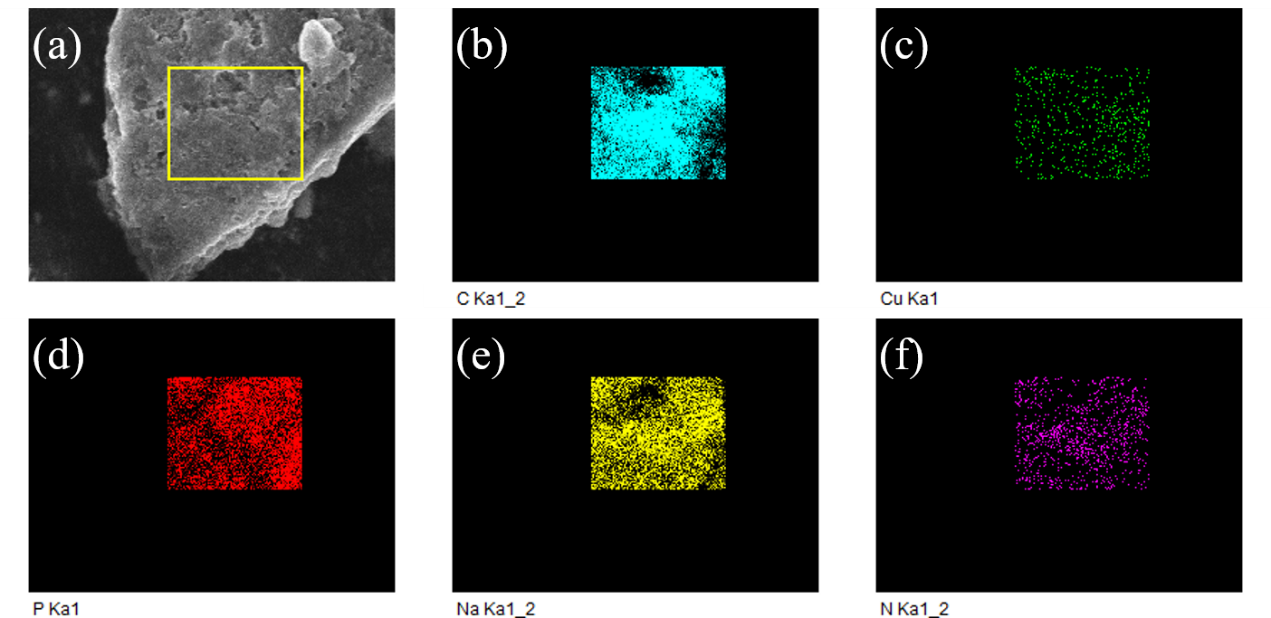


**Figure S8**. EDX mappings of selected area of Cu_3_P@P/N-C after 500 cycles.

**Table 1**. A brief summary of the typical TMPs based anode materials for SIBs

| Materials Type | Cycle stability | | | Rate capability | | Ref. |
| --- | --- | --- | --- | --- | --- | --- |
|  | Capacity  (m Ah g^-1^) | Current density  (mA g^-1^) | Cycle number | Capacity  (m Ah g^-1^) | Current density  (A g^-1^) |  |
| Cu_3_P@P/N-C | 209.3  118.2 | 1000  5000 | 1000  2000 | 207.2 | 2 | This |
|  |  |  |  | 177.1 | 5 | work |
| Cu_3_P/CNS | 150.9 | *1000* | *100* | 142.5 | 2 | Adv. Energy Mater. 2018^1^ |
| Cu_3_P nanowire | 133.8 | *1000* | *260* | 137.8 | 5 | Adv. Funct. Mater. 2016^2^ |
| Cu_3_P@C5 | 156.5 | *1000* | *1000* | 149.9 | 5 | JMCA  2019^3^ |
| Ni_2_P@NPC | 158 | *1000* | *300* | 100 | 5 | Nano Energy 2018^4^ |
| Ni_2_P⊂pGN | 161 | *200* | *100* | 101 | 2 | Adv. Mater. 2017^5^ |
| H-FeP@C@GR | 400 | *100* | *250* | 237 | 1.6 | ACS Nano 2017^6^ |
| CoP@C-RGO-NF | 473 | *100* | *100* | 115.6 | 1.6 | Nano Energy 2017^7^ |

**Reference**

1. M. Kong, H. Song and J. Zhou, *Advanced Energy Materials*, 2018, **8**, 1801489.
2. M. Fan, Y. Chen, Y. Xie, T. Yang, X. Shen, N. Xu, H. Yu and C. Yan, *Advanced Functional Materials*, 2016, **26**, 5019-5027.
3. J. Zhu, Q. He, Y. Liu, J. Key, S. Nie, M. Wu and P. Shen, *Journal of Materials Chemistry A*, 2019, **7**, 16999.
4. S. Shi, Z. Li, Y. Sun, B. Wang, Q. Liu, Y. Hou, S. Huang, J. Huang and Y. Zhao, *Nano Energy*, 2018, **48**, 510-517.
5. C. Wu, P. Kopold, P. Aken, J. Maier and Y. Yu, *Advanced Materials*, 2017, **29**, 1604015.
6. X. Wang, K. Chen, G. Wang, X. Liu and H. Wang, *ACS Nano*, 2017, **11**, 11602-11616.
7. X. Ge, Z. Li and L. Yin, *Nano Energy*, 2017, **32**, 117-124.
